# Protective effect of mussel polysaccharide on cyclophosphamide‐induced intestinal oxidative stress injury via Nrf2‐Keap1 signaling pathway

**DOI:** 10.1002/fsn3.3453

**Published:** 2023-05-23

**Authors:** Zhen‐Lei Zhao, Xiao‐Gang Xu, Yun‐Chuang Chang, Yi‐Peng Xu, Xu‐Qiang Zhou, Hui‐Li Su, Xiao‐Hua Cui, Xiao‐Qing Wan, Gen‐Xiang Mao

**Affiliations:** ^1^ Zhejiang Provincial Key Lab of Geriatrics & Geriatrics Institute of Zhejiang Province, Department of Geriatrics Zhejiang Hospital Hangzhou China; ^2^ College of Biological and Food Engineering Hubei Minzu University Enshi China; ^3^ The Key Laboratory of Zhejiang Province for Aptamers and Theranostics, Institute of Basic Medicine and Cancer (IBMC) Zhejiang Cancer Hospital, Chinese Academy of Sciences Hangzhou China; ^4^ College of Life Science Zhejiang Chinese Medical University Hangzhou China

**Keywords:** cyclophosphamide, intestinal protection, mussel polysaccharide, Nrf2‐Keap1 pathway, oxidative stress

## Abstract

The hard‐shelled mussel (*Mytilus coruscus*) has been used as a traditional Chinese medicine and health food in China for centuries. Polysaccharides from mussel has been reported to have multiple biological functions, however, it remains unclear whether mussel polysaccharide (MP) exerts protective effects in intestinal functions, and the underlying mechanisms of action remain unclear. The aim of this study was to investigate the protective effects and mechanism of MP on intestinal oxidative injury in mice. In this study, 40 male BALB/C mice were used, with 30 utilized to produce an animal model of intestinal oxidative injury with intraperitoneal injection of cyclophosphamide (Cy) for four consecutive days. The protective effects of two different doses of MP (300 and 600 mg/kg) were assessed by investigating the change in body weight, visceral index, and observing colon histomorphology. Moreover, the underlying molecular mechanisms were investigated by measuring the antioxidant enzymes and related signaling molecules through ELISA, real‐time PCR, and western blot methods. The results showed that MP pretreatment effectively protected the intestinal from Cy‐induced injury: improved the colon tissue morphology and villus structure, increased superoxide dismutase (SOD), catalase (CAT), and glutathione peroxidase (GSH‐Px) activities, and reduced malondialdehyde (MDA) content in serum and colon tissues. Meanwhile, MP also significantly increased the expression levels of SOD, GSH‐Px, heme oxygenase‐1 (HO‐1), and nuclear factor E2‐related factor 2 (Nrf2) mRNA in colon tissues. Further, western blot results showed that the expression of Nrf2 protein was significantly upregulated while kelch‐like ECH‐associated protein 1 (Keap1) was significantly downregulated by MP in the colonic tissues. This study indicates that MP can ameliorate Cy‐induced oxidative stress injury in mice, and Nrf2‐Keap1 signaling pathway may mediate these protective effects.

## Graphical abstract

Mussel polysaccharides restored and relieved the intestinal oxidative stress induced by cyclophosphamide‐induced in mice. Mussel polysaccharides can activate Nrf2 signaling pathway and upregulate antioxidant enzyme expression.

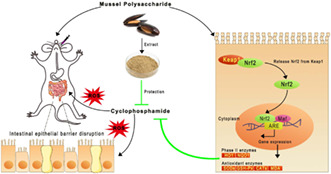

## 1 INTRODUCTION

Reactive oxygen species (ROS) are chemically reactive oxygen‐containing species, and hydrogen peroxide and superoxide anion are two examples of ROS (Sies & Jones, 2020). A high level of ROS can damage DNA, proteins, and lipids if it is not quenched immediately, and this damage is termed oxidative stress (Martin et al., 2020). Excessive oxidative stress provokes a wide variety of disorders, including cell dysfunction and apoptosis, organism aging, and the occurrence of multiple chronic diseases (Ortega et al., 2021; Pizzino et al., 2017; Shen et al., 2017). The intestine is an essential organ for digestion, absorption, and secretion (Gong et al., 2021). However, as the gateway to the outside world, the intestinal is easily impacted by the surrounding environment, especially the oxidative stress conditions. Oxidative stress plays an important role in intestinal barrier disruption, which will lead to increased infiltration of toxins. It is possible that excessive oxygen free radical accumulation will destroy the mucosal barrier, increase intestinal permeability, and disrupt the intestinal flora, thereby destroying the body's steady state (Turan et al., 2017; Xue et al., 2020). Several studies showed that intestinal barrier dysfunction is associated with diverse chronic disorders, such as inflammatory bowel disease, obesity, diabetes, and rheumatologic diseases (Ramos & Papadakis, 2019). Therefore, many studies have focused their attention on finding substances that could counter intestinal oxidative stress injury.

Natural polysaccharides, an important bioactive substance, are widely existed in plants, animals, microorganisms, and marine organisms. Polysaccharides have drawn greater attention for their health benefits and exhibit great structural variability and the ability to hold biological information (Gan et al., 2021). Polysaccharides have various biochemical functions, including antioxidant (Zhou et al., 2021), antiviral (Wang et al., 2021), anti‐inflammatory (Xiang et al., 2021), immune regulation (Ruijun et al., 2015), and antitumor effects (Ye et al., 2021). The hard‐shelled mussel (*Mytilus coruscus*) is a kind of marine shellfish with homology of food and medicine, which mainly grows in the shallow sea between the rocks (Chen et al., 2019). It is widely distributed in the Bohai Sea and the Yellow Sea of China. Owing to its high nutritional value and good medicinal potential, mussels have been widely artificially farmed throughout the world and have a high yield (Li et al., 2020). According to previous studies, mussel polysaccharide (MP) is the main active ingredient for its nutritional and medicinal values (Liu et al., 2017). Particularly, MP has been reported to have significant antioxidant, free radical scavenger, and immunomodulatory effects. Xiang et al. found that MP had a certain scavenging ability, reducing ability, and strong anti‐lipid peroxidation ability on hydroxyl free radical and superoxide anion free radical in vitro (Umasuthan et al., 2012). In 2021, Xiang et al. further examined the anti‐inflammatory and antioxidant activities of MP on RAW264.7 cells and DSS‐induced colitis mouse model, their results found that the MP was able to reduce inflammation and relieve some clinical symptoms of colitis (Xiang et al., 2021). A review article also summarized the current understanding of the potential health benefits of MP, discussed several studies that have demonstrated the antioxidant and anti‐inflammatory properties of the polysaccharide, as well as its potential applications in the prevention and treatment of various diseases related to oxidative stress and inflammation (Liu et al., 2021). In addition, MP was also found can boost immune activity in mice (Xiang, Wang, Chen, Chen, Zheng, et al., 2022). It is well‐known that the biological activities of polysaccharides as complex biomolecules are closely related to their composition, molecular size, and conformational shapes (Yao et al., 2021). A recent study has revealed the structure of MP (Xiang, Wang, Chen, Chen, Shen, et al., 2022), which is mainly composed of glucose, with a small amount of galactosamine hydrochloride, glucosamine hydrochloride, and galactose. The main chain connection mode of MP was determined to be →4)‐α‐d‐Glcp‐(1→glycosidic linkage, while the terminal group of α‐d‐Glcp‐(1→and α‐d‐Glcp‐(1→6)‐α‐d‐Glcp‐(1→pass→4,6)‐α‐d‐Glcp‐(1→ was bonded to the main chain via O‐6. However, there are few studies investigating its protective action for the intestinal in vivo, and the exact mechanism of action is also needed to identify.

Cyclophosphamide (Cy) is a widely used agent in the treatment of systemic autoimmune diseases and cancer (Emadi et al., 2009; Pilz et al., 2008), including germ cell tumors, sarcomas, lymphoma, and lung cancer. However, Cy has a variety of side effects according to clinical studies, such as impairing ovarian and fertility function, decreasing immune function, and causing excessive production of oxygen free radicals which in turn leads to gastrointestinal mucosal barrier damage (Shi et al., 2017; Xue et al., 2020). Therefore, the present study established the intestinal oxidative damage mouse model by intraperitoneal injection of Cy and sought to investigate the protective effect of MP on Cy‐induced intestinal oxidative injury and elucidate the underlying molecular mechanisms of its protective actions. This study provided evidence that MP can be used in food and pharmaceuticals as natural intestinal antioxidant protection.

## 2 METHODS

### 2.1 Animals and study design

In this study, the SPF BALB/C mice (8‐week‐year old, weighted 18–22 g) were obtained from Shanghai Slack Laboratory Animal Co., LTD (Shanghai, China). Animal experiments were all performed according to the Institutional Animal Care and Use Committee (IACUC) of Zhejiang University of Technology (Approval No. 201907). The animal care was conducted according to institutional guidelines. A controlled environment was used to house the mice under a 12 h light/dark cycle and had free access to water and food.

The mice were randomly divided into four groups (*n* = 10) after 1 week of adaptive feeding, the detailed grouping information is shown in Table 1. Each group received 21 consecutive days of intragastric administration of MP or vehicle. From day 18 to 21, Cy was given through intraperitoneal injection for 4 days. Each week, food intake and body weight were recorded during the experiments. Finally, the blood and tissues were collected after the mice were fully anesthetized and sacrificed. Tissues were snap‐frozen in liquid nitrogen and stored at −80°C until assay.

**TABLE 1:** Animal grouping.

Groups	Stage I (1–21th days, MP)	Stage II (18–21th days, Cy)
N	Deionized water	Saline
Y	Deionized water	Cy (50 mg/kg∙BW)
L‐MP	MP (300 mg/kg∙BW)	Cy (50 mg/kg∙BW)
H‐MP	MP (600 mg/kg∙BW)	Cy (50 mg/kg∙BW)

Abbreviations: H‐MP, mussel polysaccharides high‐dose administration group; L‐MP, mussel polysaccharides low‐dose administration group; N, normal group; Y, model group.

### 2.2 Chemicals and reagents

The standard feed for mice was purchased from Beijing Keaoki Feed Co. Ltd. (Beijing, China). Cyclophosphamide was obtained from Sigma Aldrich Company (St Louis, USA). SOD, GSH‐Px, CAT, and MDA assay kits were purchased from the Nanjing Jiancheng Institute of Biological Engineering (Nanjing, China). The Nrf2 and Keap1 antibodies for western blot were purchased from the Abcam (Cambridge, MA, USA). Rabbit anti‐GAPDH and hematoxylin and eosin (HE) staining solutions were purchased from Beijing Solaibao Technology Co. Ltd (Beijing, China). In addition to the above reagents, all other analytical reagents are either of the highest or commercial grade.

### 2.3 Preparation of mussel polysaccharide

Fresh mussels were purchased from the Hangzhou Seafood Market, Zhejiang Province. Mussel polysaccharide (MP) was extracted and prepared by Dr. Xiang's laboratory in Zhejiang University of Technology, the method was described previously (Xiang, Wang, Chen, Chen, Shen, et al., 2022). Briefly, the fresh mussel flesh and its soft tissues were collected and homogenized immediately. Then the homogenized solution was degreased by the Soxhlet extraction method and then dissolved in distilled water, and 1% papain and 1% alkaline protease were added, respectively. After extraction, the enzyme was removed at 99°C for 20 min, cooled to room temperature, and adjusted the pH value to neutral. Centrifuged at 3000× *g* for 15 min, the supernatant was taken and the protein was removed by the Sevage method (Zhu et al., 2017) (the volume ratio of chloroform to n‐butanol was 4:1) to obtain the extract. The supernatant was concentrated to 1/3 of the original volume by rotary evaporator and was sunk with 95% ethanol, four times the volume, and placed in a refrigerator at 4°C overnight. The next day, the precipitation was taken from the centrifugal solution and freeze‐dried. The dried sample was mussel polysaccharide.

### 2.4 Measurement of visceral index

In this study, all mice were sacrificed after 21 days' treatment. Viscera index of the main organs, including the liver, spleen, and thymus were isolated from the treated and control mice to evaluate the toxicity of Cy and the protective effects of MP. The liver, spleen, and thymus were taken and weighted, the organ index was calculated as follows:

(1) $$\text{Organ index}\;\left(\%\right)=\text{organ weight}\;\left(\mathrm{mg}\right)/\text{body weight}\;\left(g\right)\times 100$$

### 2.5 Histomorphological observation of colon tissues

Mice were sacrificed with posterior neck dislocation and then dissected immediately. The whole section of the node tissue from the cecal node to the anus was removed, and the colon was quickly cut open along the mesentery direction, and the intestinal contents and residual blood were rinsed with normal saline, and then dried with clean filter paper. Fixed colon tissues were dehydrated in graded ethanol for 45 min, followed by paraffin‐embedded sections and HE staining. The dried HE sections were placed into an optical microscope for observation.

### 2.6 Detection of antioxidant factors in serum of mice

One milliliter of blood was taken from the abdominal aorta and placed in a clean tube. The serum was centrifuged at 4°C and 3000× *g* for 10 min. SOD, GSH‐Px, CAT, and MDA activities in serum were determined according to kit instructions.

### 2.7 Detection of antioxidant factors in colon tissue

One hundred milligrams of colon tissues was taken and homogenized in a tissue grinder with 1 mL 0.9% saline on ice. The supernatant was centrifuged at 3000× *g* for 10 min at 4°C, and the contents of antioxidant enzymes SOD, GSH‐Px, CAT, and MDA in colon homogenate were determined according to the production instructions of commercial kits.

### 2.8 Quantitative real‐time reverse transcription PCR

A reverse transcription kit (TIANGEN Biotech Co. Ltd., Beijing, China) was used to reverse transcribe total RNA from colon tissues into cDNA and qRT‐PCR was used to determine the levels of mRNA. The primers were designed with Primer Premier 6.0 software and synthesized by Shenggong Bioengineering Technology Limited (Shanghai, China). The sequences of primers used can be found in Table 2. A Roche LightCycler 480 II RT‐PCR Detection System was used for all quantitative real‐time PCR reactions. Gene expression was quantified using the 2^−ΔΔ*Ct*^ method (Livak & Schmittgen, 2001). The housekeeping gene β‐actin was used as a control.

**TABLE 2:** Sequences used for qRT‐PCR.

Genes	Primer sequence (5′ to 3′)		Gene number
*SOD*	Forward	AGCCAAATCCTTTCTCTCCAG	NM_011434
	Reverse	CACCGTGTTCTTCGACATCA	
*GPX*	Forward	GCTACTGTGCCGTGTGCAA	NM_008160.6
	Reverse	TGTCAATGGTGCATTGGTTTG	
*Nrf2*	Forward	GCCAACCTCCTGATGCTTCT	NM_010902
	Reverse	TCGTACACCGGGACCACAT	
*HO‐1*	Forward	GATAGAGCGCAACAAGCAGAA	NM_010442.2
	Reverse	CAGTGAGGCCCATACCAGAAG	
*NQO1*	Forward	GGACATGAACGTCATTCTCT	NM_008706.5
	Reverse	TTCTTCTTCTGCTCCTCTTG	
*β‐Actin*	Forward	AGTGTGACGTTGACATCCGT	NM_007393
	Reverse	GCAGCTCAGTAACAGTCCGC	

### 2.9 Western blot analysis

RIPA lysis buffer with protease and phosphatase inhibitor cocktail was used to homogenize colon tissues. After centrifugation at 12,000× *g* for 15 min at 4°C, tissue debris was removed. Then, the total protein was quantified by BCA quantitative kit (Solaibao, Beijing, China) and diluted to 2–4 mg/mL. Equal amounts of total proteins were electrophoresed. Afterward, the gel was transferred to a 0.45 μm PVDF membrane (Millipore). Then, the membrane was blocked with TBST (Tris‐buffered saline with 0.05% Tween‐20) containing 5% skim milk powder for 2 h. The membranes were then incubated at 4°C with the primary antibody overnight. Subsequently, the membranes were washed 5 min with TBST for three times, and then, HRP‐conjugated secondary antibodies were incubated for 1 h at room temperature. Finally, after rewashing the membranes with TBST for three times, images of the protein bands were obtained using electrochemiluminescence (ECL).

### 2.10 Statistical analysis

The figure legends for each experiment provide all the statistical information, including the statistical tests used, number of mice (represented as *n*) in the animal experiments. Date were expressed as mean ± standard deviation (SD), and the Student's *t*‐test was adopted to analyze the significant differences between the two groups using GraphPad Prism 6.0 (GraphPad Software, Inc.), and significant differences were designated as *p* < .05.

## 3 RESULTS

### 3.1 Mussel polysaccharides increase the body weight gain and organ index of mice

**FIGURE 1:**
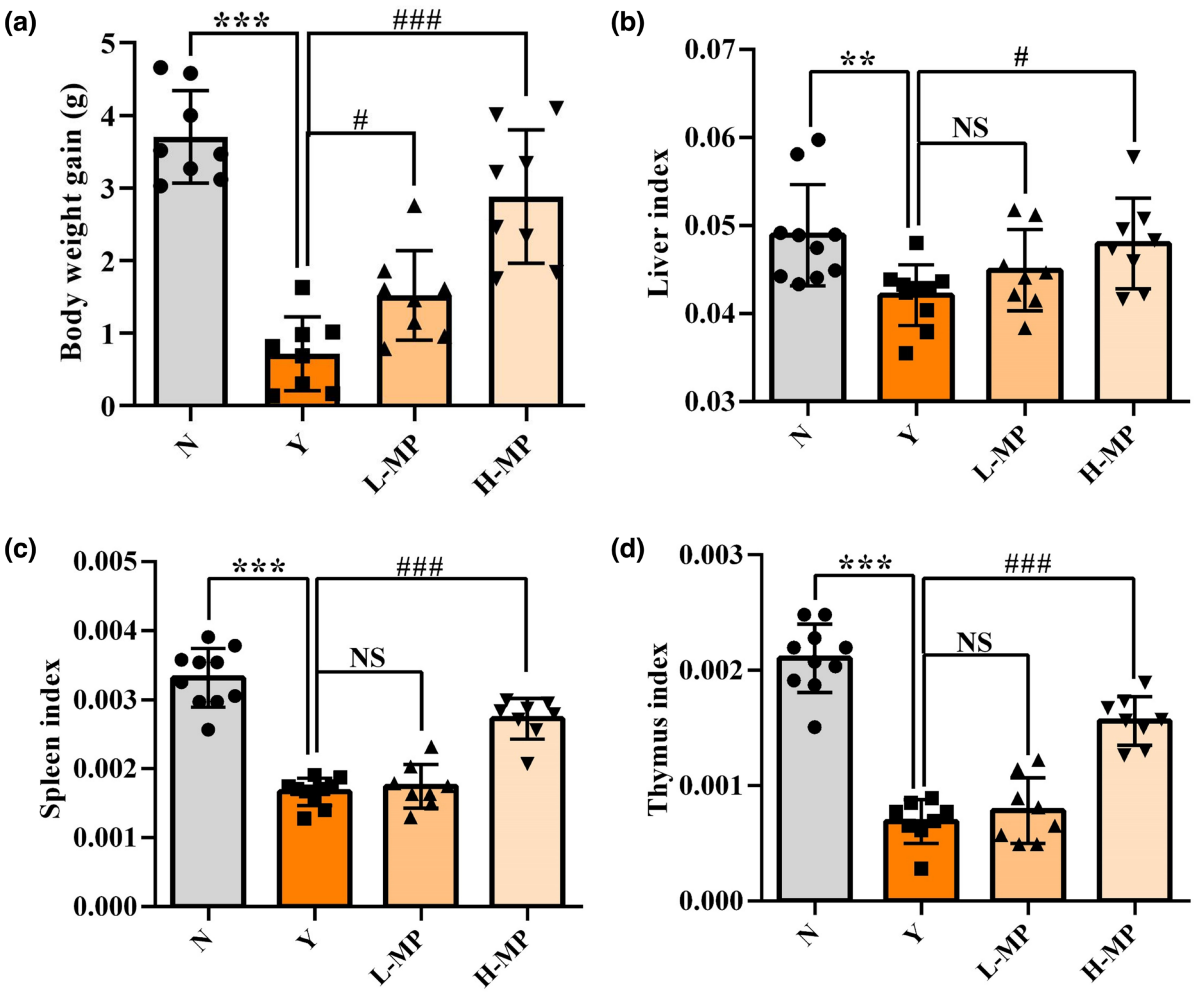
Effect of mussel polysaccharide on average daily gain and organ index in oxidized mice. (a) average daily gain, (b) liver index, (c) spleen index, (d) thymus index. *n* = 8–10 mice per group, the mean values ± SD are presented, **p < .01, ***p < .001, N group versus Y group; NS: not significant, ^#^ *p* < .05, ^###^ *p* < .001, L‐MP or H‐MP versus Y group.

Figure 1 shows the body weight gain and organ index (liver, spleen, and thymus index) of mice during the 21‐day treatment. Cy treatment significantly reduced the body weight gain and organ index of mice compared with the normal group (*p* < .05). Compared with the Cy‐treated model group, the body weight gain (Figure 1a), liver index (Figure 1b), spleen index (Figure 1c), and thymus index (Figure 1d) were all obviously increased by advanced intragastolic administration of MP in a dose‐dependent manner, and the high‐dose MP group showed a significant difference in comparison with the model group (*p* < .05). The body weight gain and organ index (liver, spleen, and thymus index) in low‐dose MP group were also increased, but there was no significant difference (*p* > .05).

### 3.2 Mussel polysaccharides protect colon tissues from oxidative stress in mice

**FIGURE 2:**
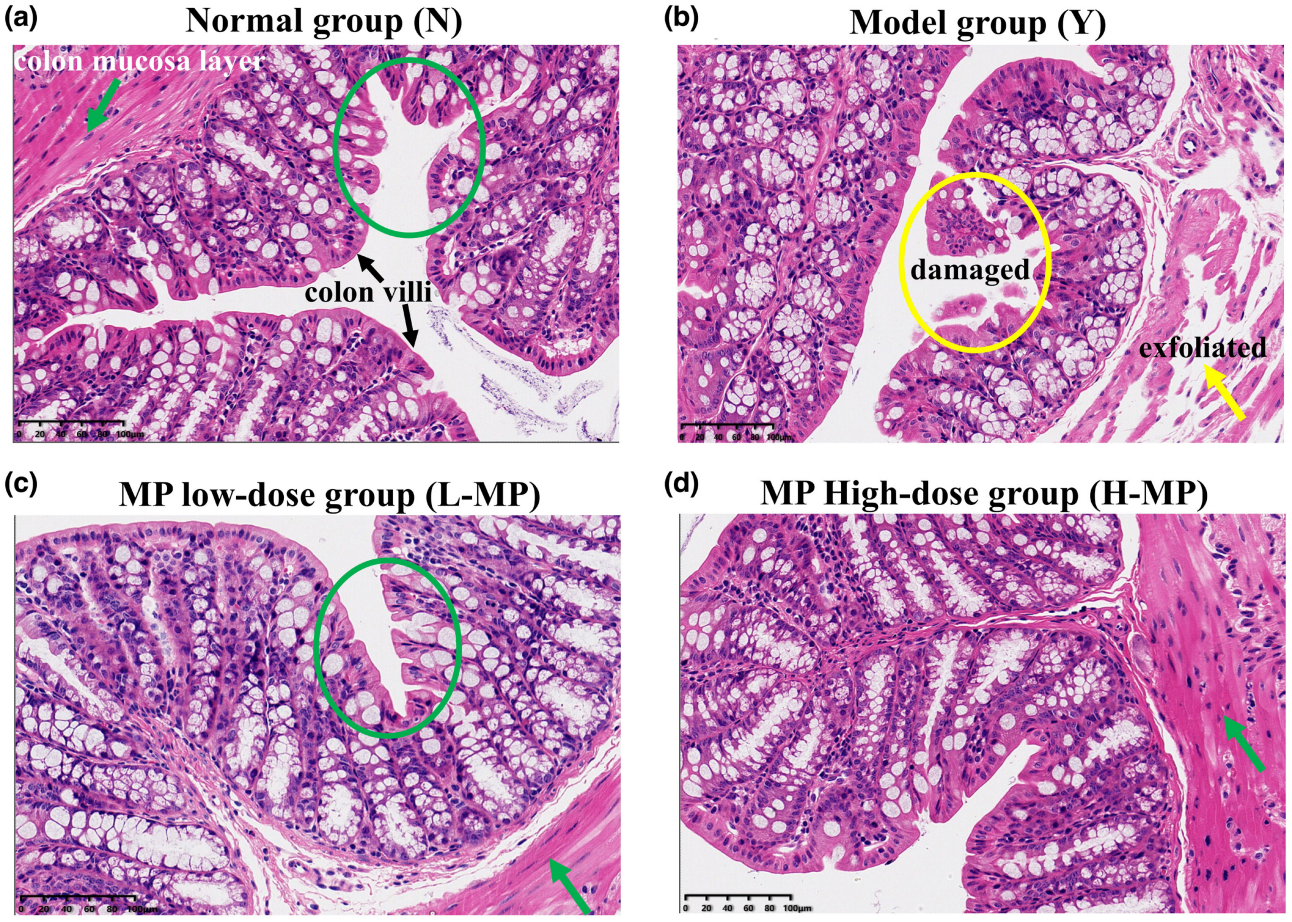
Representative colonic sections were stained with HE to visualize morphological structures in (a) normal group, (b) Cy‐treated model group, (c) MP low‐dose group, and (d) MP high‐dose group. The blue ellipses indicate normal histological structure of colon villi, the yellow ellipses represent damaged colon villi histological structure, the green arrow represents complete colon mucosa layer, and the yellow arrow represents damaged and exfoliated colon mucosa layer.

Effects of MP on the colonic morphology of mice were investigated. HE staining results are shown in Figure 2. The intestinal villi of the normal group were arranged in an orderly, slender, and compact manner, the upper epidermis of the colon mucosa was complete, and the mucosa layer was rich in glands with uniform structure. The cup cells in the glands of the mucosa layer were not shed, and the intestinal wall was thick (Figure 2a). However, the intestinal villi of the Cy‐treated model group exhibited incomplete structure, with the irregular arrangement, loose and short, and some villi were damaged and exfoliated (Figure 2b). The upper epidermis of the colon mucosa layer was largely exfoliated, and the glands in the colon mucosa layer were partially cracked and disappeared. In contrast, in the MP pretreatment group, the villi length was restored, and the structure was fairly complete, the arrangement was relatively orderly, and the damaged state was improved, as shown in Figure 2c,d. Results above revealed that earlier intake of MP effectively protected colon tissues from Cy‐induced oxidative stress and reduced intestinal villi injury.

### 3.3 Mussel polysaccharides improved serum antioxidant enzyme levels

**FIGURE 3:**
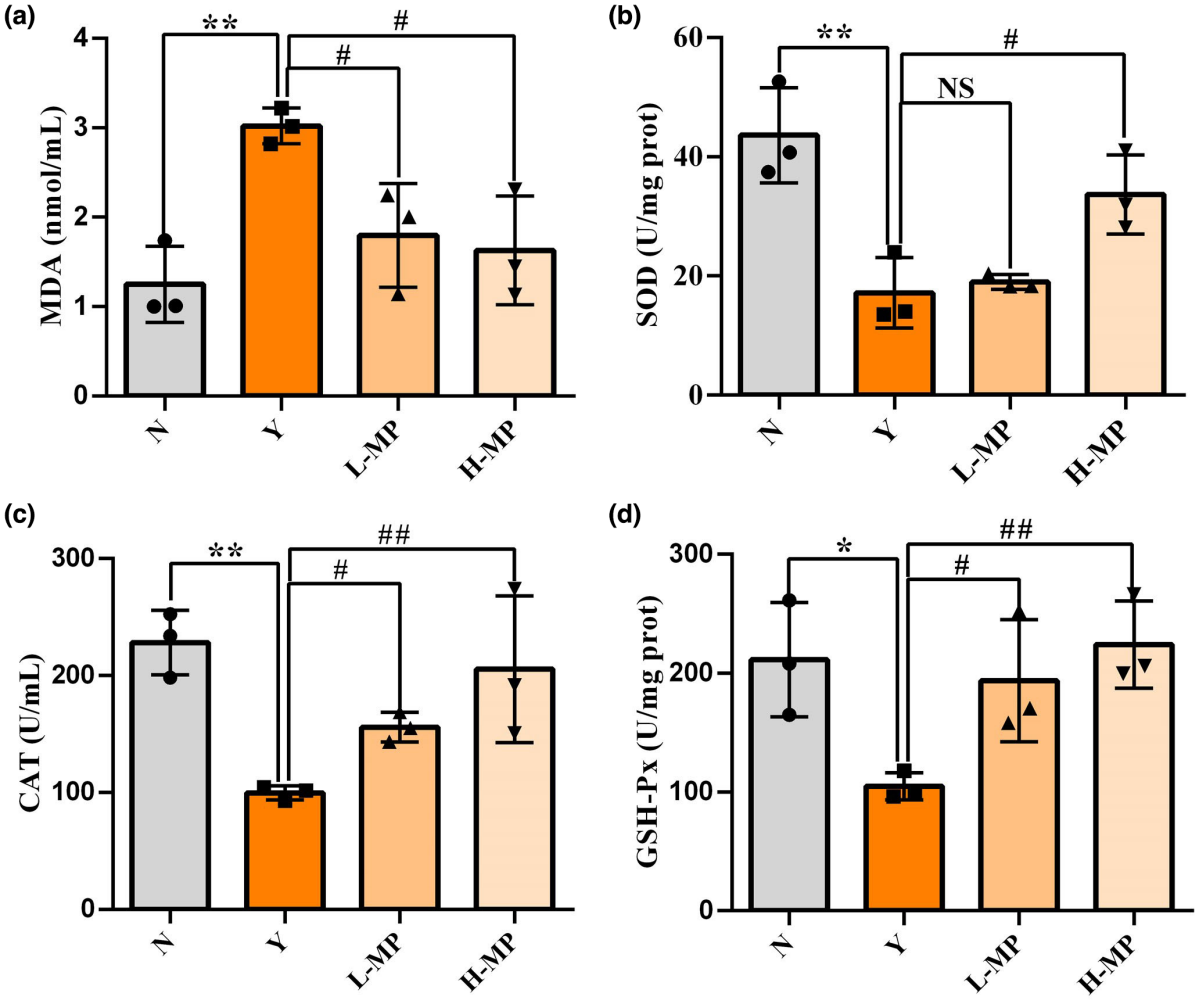
Effect of mussel polysaccharide on serum oxidative stress indicators (a) MDA, (b) SOD, (c) CAT, and (d) GSH‐Px in mice. *n* = 3 mice per group, the mean values ± SD are presented, *p < .05, **p < .01, N group versus Y group; NS: not significant, ^#^ *p* < .05, ^##^ *p* < .01, L‐MP or H‐MP versus Y group.

MDA, SOD, CAT, and GSH‐Px are four important oxidative stress indicators (Wu et al., 2019). SOD is a widespread free radical scavenger in vivo, which plays an important role in alleviating oxidative stress state in vivo (Gupta et al., 2009). MDA is an aldehyde produced by lipid peroxidation, which can reflect the degree of peroxidation in the body (Fois et al., 2020). GSH‐Px is a good antioxidant in the body, which can scavenge and neutralize free radicals and peroxides in the body (Maher, 2005). CAT as an antioxidant enzyme can transform H_2_O_2_ into H_2_O and O_2_, thus playing an important role in scavenging free radicals (Dai et al., 2021). As shown in Figure 3, serum MDA content (Figure 3a) in the model group was higher than that in the normal group (*p* < .05), while SOD (Figure 3b), CAT (Figure 3c), and GSH‐Px (Figure 3d) contents were both lower than those in normal group (*p* < .05), indicating that the Cy‐induced oxidative stress model is successfully established. In comparison with the Cy‐treated model group, MP intervention not only effectively decreased MDA content, but also increased SOD, CAT, and GSH‐Px levels in mice serum, especially for the high‐dose MP group, a significant difference (*p* < .05) was observed. Meanwhile, both the four indicators of the high‐dose MP group showed no obvious difference compared with the normal group (*p* > .05). The above results showed that MP enhanced antioxidant capacity of mice and improved the serum oxidative stress induced by Cy effectively.

### 3.4 Mussel polysaccharides enhanced antioxidant capacity in colon tissues

**FIGURE 4:**
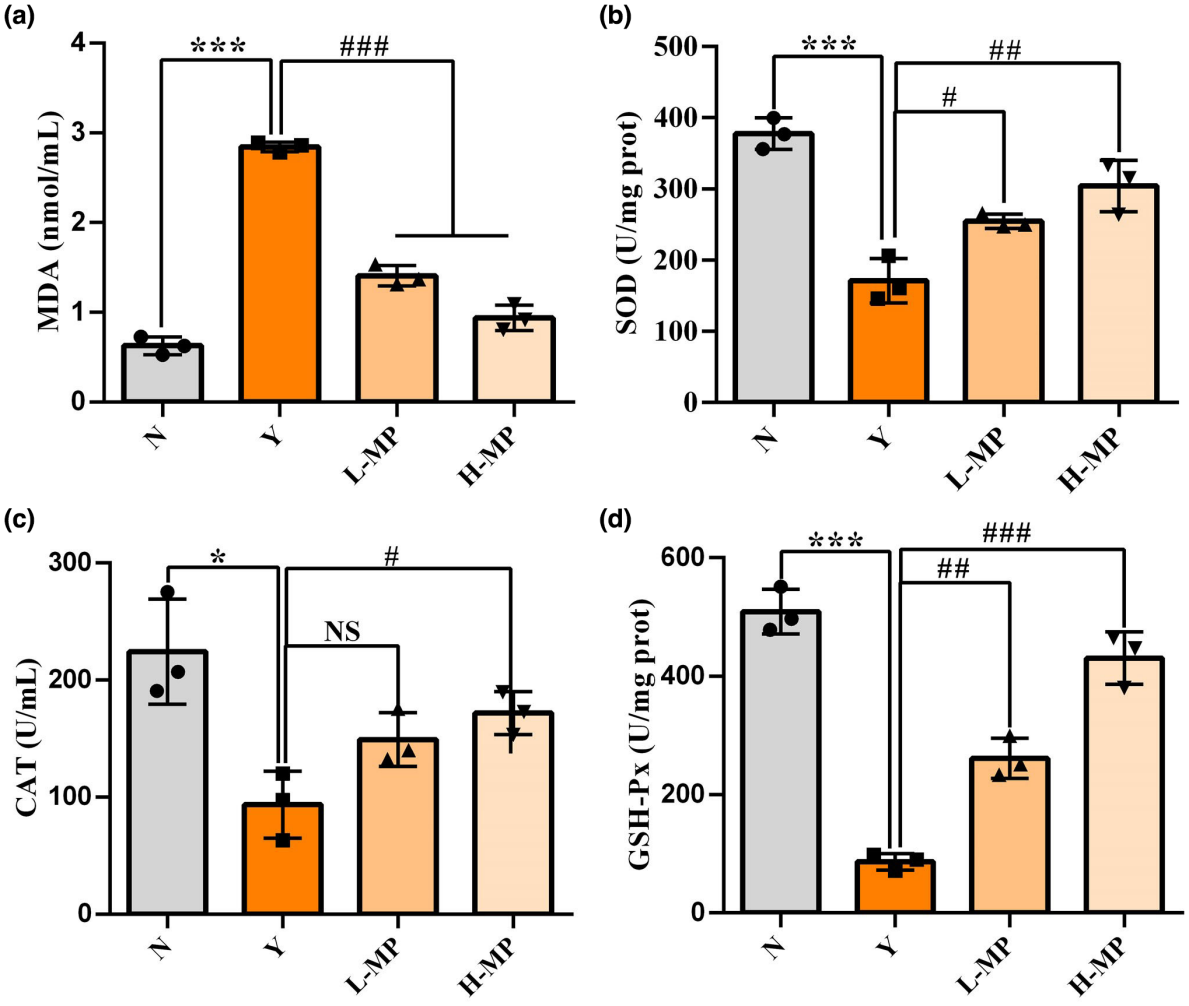
Effect of mussel polysaccharide on antioxidant levels of (a) MDA, (b) SOD, (c) CAT, and (d) GSH‐Px in mice colon tissues. *n* = 3 mice per group, the mean values ± SD are presented, *p < .05, ***p < .001, N group versus Y group; NS: not significant, ^#^ *p* < .05, ^##^ *p* < .01, ^###^ *p* < .001, L‐MP or H‐MP versus Y group.

The effect of MP on the antioxidant levels in colon tissues was evaluated, results can be found in Figure 4. Compared with the normal group, Cy significantly elevated the MDA levels (Figure 4a, *p* < .05) and decreased SOD (Figure 4b), CAT (Figure 4c), and GSH‐Px (Figure 4d) activities in mice colon tissues. Compared with Cy‐treated modeling group, MP pretreatment elevated both the contents of SOD, CAT, and GSH‐Px, and decreased the levels of MDA in a dose‐dependent manner in the colon tissues. The results showed that the low‐dose MP could exert improved effects on MDA, SOD, and GSH‐Px except for CAT. And for the MP high‐dose‐treated groups, significant between‐group differences were observed in all indexes (*p* < .05). The results revealed that pretreatment of MP effectively enhanced the antioxidant capacity of mice and alleviated Cy‐induced oxidative stress damage in a dose‐dependent manner.

### 3.5 Effect of mussel polysaccharides on antioxidation‐related genes in colon tissues

**FIGURE 5:**
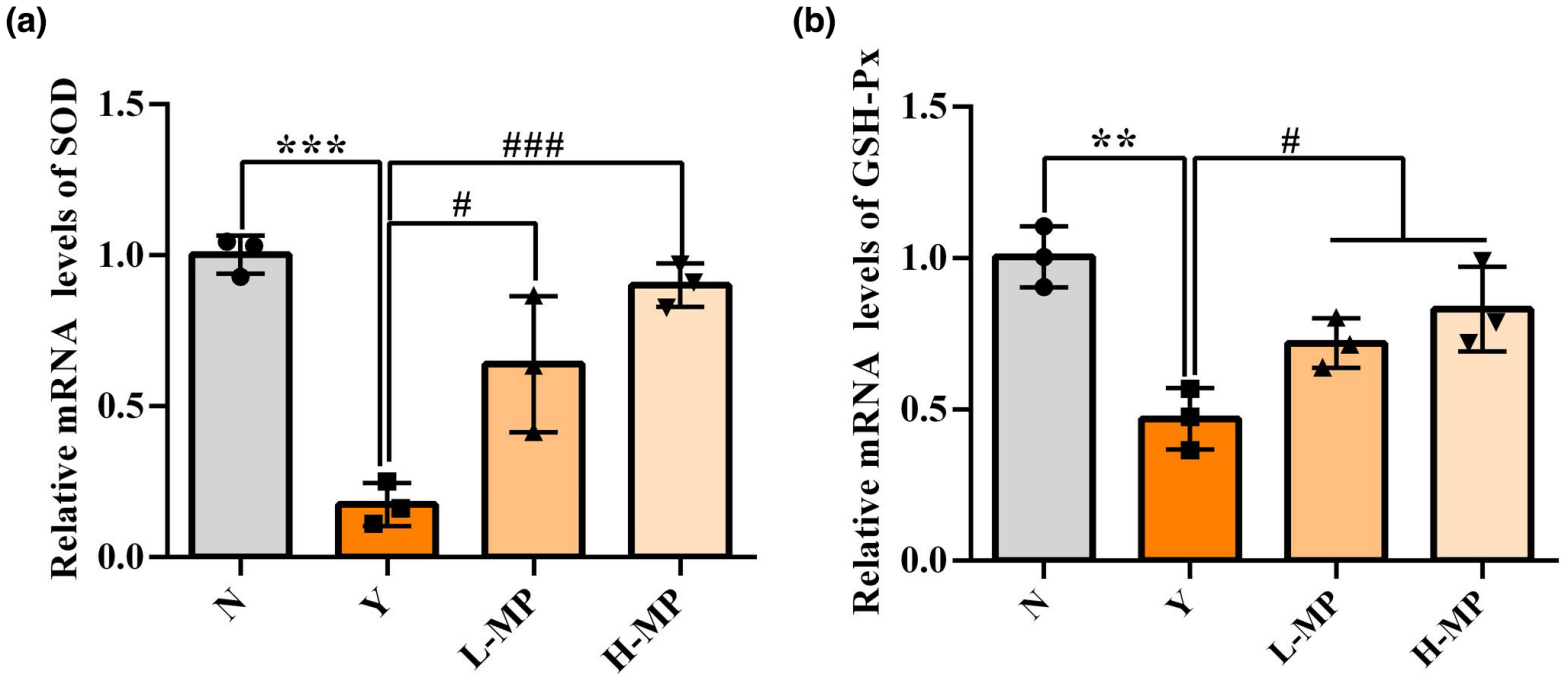
Effect of mussel polysaccharide on the relative expression levels of antioxidant genes (a) SOD and (b) GSH‐Px in mice colon tissues. *n* = 3 mice per group, the mean values ± SD are presented, **p < .01, ***p < .001, N group versus Y group; ^#^ *p* < .05, ^###^ *p* < .001, L‐MP or H‐MP versus Y group.

Effects of MP on the related antioxidant gene expressions in mice colon tissues were evaluated, results are shown in Figure 5. In comparison to the normal group, Cy obviously reduced the mRNA expression levels of both SOD (Figure 5a) and GSH‐Px (Figure 5b) in colon tissues (*p* < .05). Compared with the Cy‐treated model group, the mRNA expression levels of SOD and GSH‐Px were all obviously elevated by pretreatment of MP (*p* < .05) in a dose‐dependent manner. The above results reveal that MP appears to regulate antioxidant genes expression and rescue Cy caused intestinal antioxidant capacity reduction.

### 3.6 Effect of mussel polysaccharides on Nrf2 and Nrf2‐modulated phase II detoxifying gene expression levels in colon tissues

**FIGURE 6:**
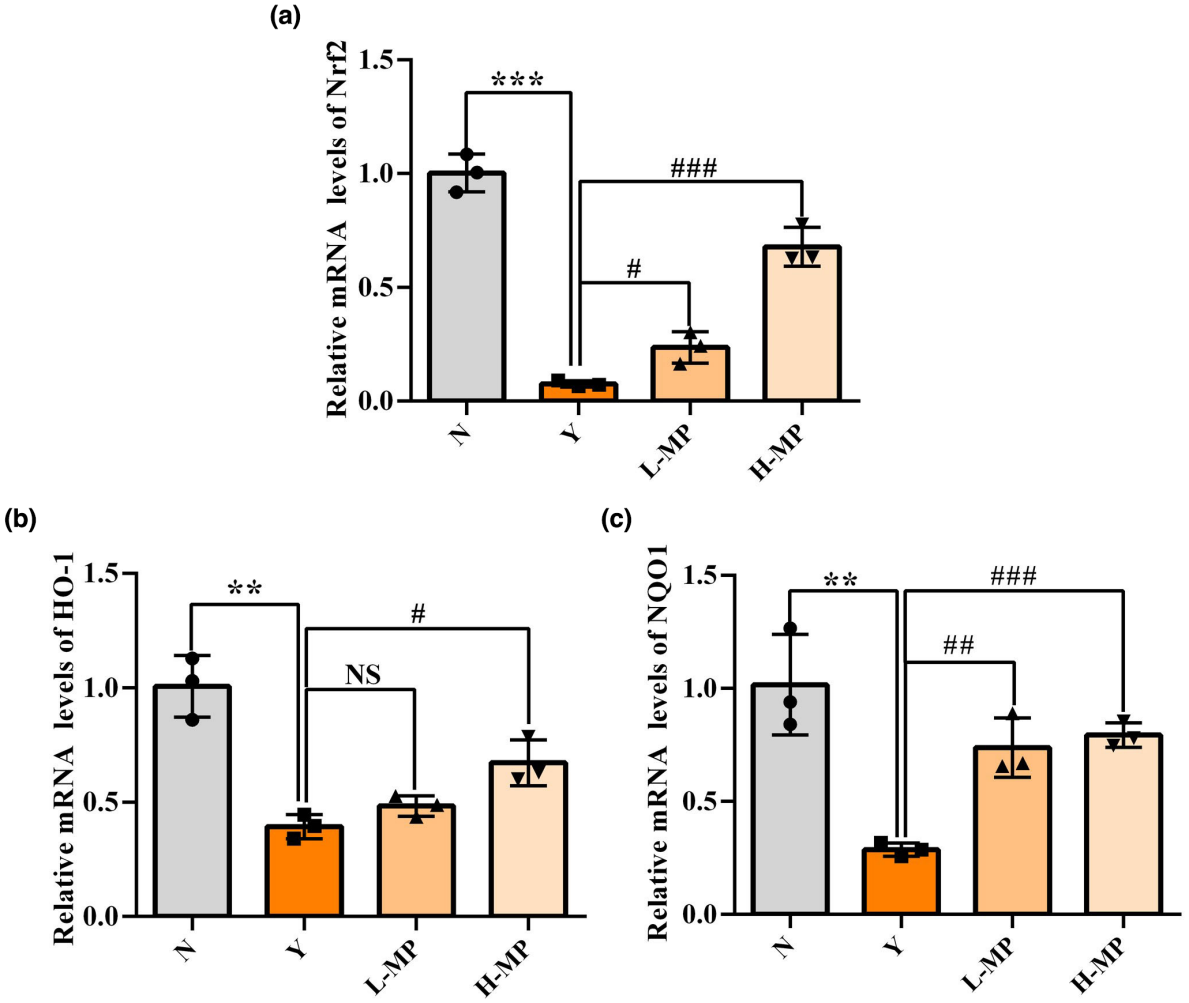
Effect of mussel polysaccharide on the relative gene (a) Nrf2, (b) HO‐1, and (c) NQO1 expression at mRNA levels in mice colon tissues. *n* = 3 mice per group, the mean values ± SD are presented, **p < .01, ***p < .001, N group versus Y group; NS: not significant, ^#^ *p* < .05, ^##^ *p* < .01, ^###^ *p* < .001, L‐MP or H‐MP versus Y group.

As an important antioxidant protein, the mRNA expression levels of Nrf2 and its driving detoxifying genes HO‐1 and NQO1 were evaluated, results are indicated in Figure 6. Compared with the normal group, the Nrf2 expression levels were significantly decreased by intraperitoneal injection of Cy (Figure 6a, *p* < .001). And as expected, Nrf2 downregulation led to attenuated gene expression of its target genes HO‐1 (Figure 6b) and NQO1 (Figure 6c). Compared with the Cy‐treated model group, both the low‐ and high‐dose MP promoted both the Nrf2, HO‐1, and NQO1 gene expressions in mice colon tissues, and a dose‐dependent effect was observed. These results indicate that MP may protect the intestinal from Cy‐induced oxidative stress injury via Nrf2 signaling to activate antioxidant systems.

### 3.7 Effect of mussel polysaccharides on Nrf2‐Keap1 signaling pathway

**FIGURE 7:**
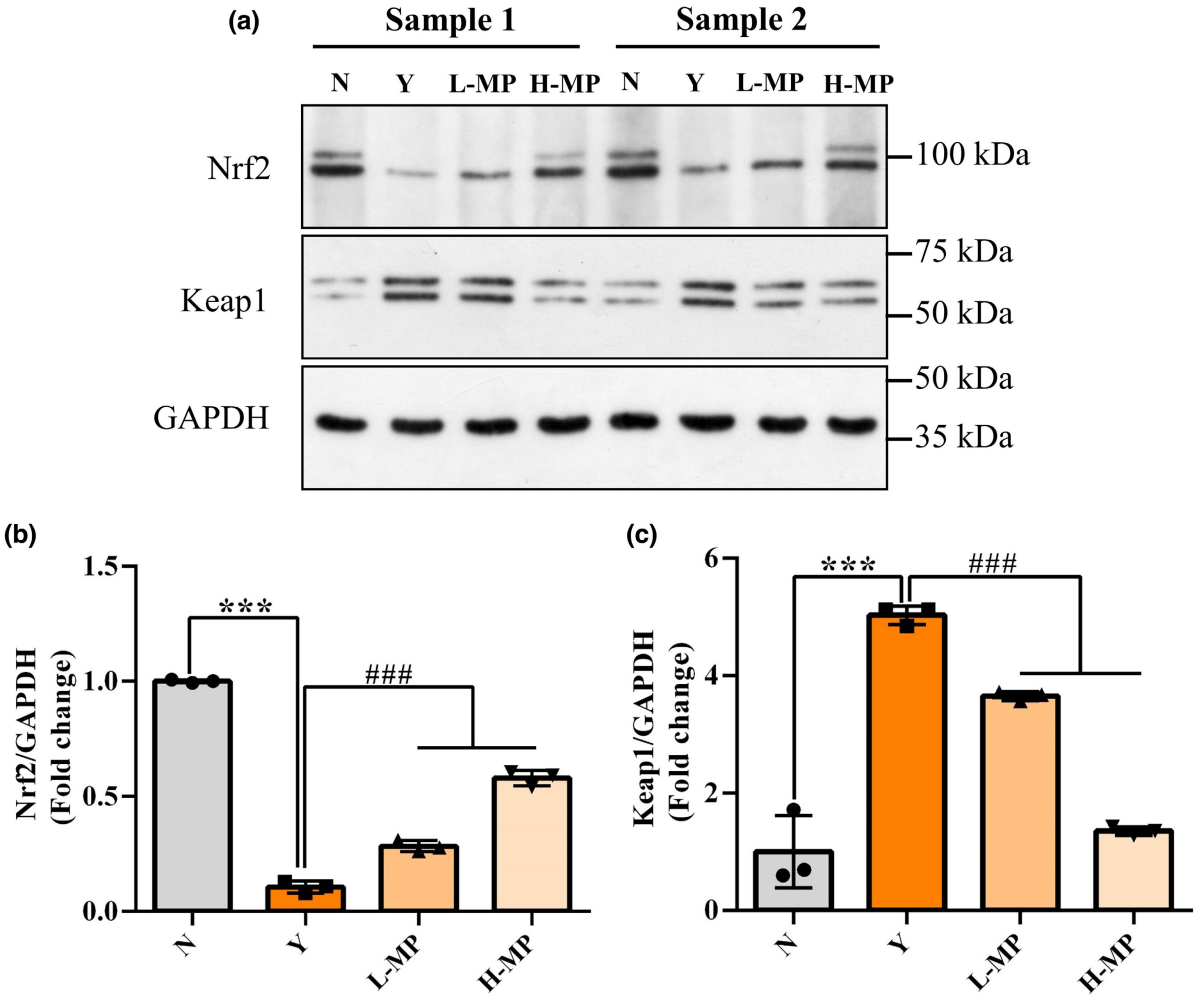
Effect of mussel polysaccharide on Nrf2‐Keap1 signaling pathway in mice colon tissues. (a) Western blot analysis of Nrf2, Keap1, and GAPDH protein expression levels in the whole colon tissue lysates. (b) Nrf2 and (c) Keap1 protein expression levels, quantified by determining the gray value. Representative images and densitometries from three independent experiments are shown. *n* = 3 mice per group, the mean values ± SD are presented, ***p < .001, N group versuss Y group; ^###^ *p* < .001, L‐MP or H‐MP versus Y group.

It is well known that activation of the Nrf2–Keap1 signaling pathway plays a critical role in protecting cells from oxidative stress damage. Western blot analysis was used to evaluate the effect of MP on Nrf2‐Keap1 signaling pathway, Nrf2 and Keap1 protein expression levels present in Figure 7. As compared to the normal group, Cy greatly lowered Nrf2 protein expression (Figure 7a,b), while elevated Keap1 protein expression (Figure 7a,c) in the colon tissues (*p* < .05). Compared with the Cy‐treated modeling group, MP pretreatment observably promoted the expression of the Nrf2 protein (*p* < .05), and at the same time remarkably inhibited the Keap1 expression levels (*p* < .05), displaying a good dose‐dependence. These results further proved that the activation of Nrf2‐Keap1 signaling pathway plays a critical role in protecting the intestine against oxidative stress induced by Cy in mice.

## 4 DISCUSSION

Mussels are considered a high‐quality food choice for consumers due to their nutritional value. According to previous reports, the nutritional value of mussels could be attributed to the presence of high levels of polysaccharides (Chen et al., 2019; Xiang, Wang, Chen, Chen, Shen, et al., 2022). In recent years, polysaccharides isolated from traditional Chinese medicines have attracted substantial attention due to their various bioactivities and low toxicity. Numerous studies have shown that polysaccharides possess wide‐ranging beneficial therapeutic effects and health‐promoting properties including antitumor, antiviral, inflammation modulation, and antioxidant activities (Tadayoni et al., 2015; Vanavil et al., 2020; Wang et al., 2021). Wong et al. showed that the protective effects of *Angelica sinensis* polysaccharides on ulcerative colitis in rats are closely related to the prevention of oxidative stress and lipid peroxidation in the colon (Wong et al., 2008). Huang et al. reported that *Ligusticum chuanxiong*‐derived pectin polysaccharide (LCP‐II‐I) enhanced the antioxidant defense of aged mice intestines (Huang et al., 2017).

Our recent work characterized MP's structure and evaluated its antioxidant capacity on RAW264.7 cells (Xiang, Wang, Chen, Chen, Shen, et al., 2022). In view of the obvious anti‐inflammatory activities of MP in vitro, we further carry out animal experiments to investigate its beneficial effect in vivo. Cy as a common clinical chemotherapy drug, has antitumor effect, which is also used to treat autoimmune diseases. However, the active alkylating compounds of Cy in vivo (4‐hydroxy‐cyclophosphamide and aldophosphamide) will combine with macromolecules in the cells and change the important functional enzyme activities, resulting in an imbalance between the internal and external environment of the cells, and inducing oxidative stress damage, and eventually cause damage to the entire organization and function of the cell (Senthilkumar et al., 2006; Shi et al., 2017; Wen et al., 2019). The present study established an acute intestinal oxidative stress damage model intraperitoneal injection of Cy and investigated the protective effects and the underlying mechanisms of MP on intestinal mucosal oxidative injury by early administration of MP.

In the antioxidant defense system, SOD, GSH‐Px, and CAT play crucial roles. SOD can scour free radicals, reduce lipid peroxidation and maintain the balance between oxidation and antioxidant in the body. As a result of GSH‐Px and CAT, free radicals can be effectively removed from the body, preventing damage caused by free radicals. A marker of oxidative stress, MDA is the result of lipid peroxidation, which may cause DNA rearrangement and cell apoptosis. In addition to digestion and absorption of nutrients, the intestinal tract is the body's largest immune system. Thus, this study also measured antioxidant levels in colon tissues. HE staining results suggested that the intestinal tissues of mice in the Cy‐treated modeling group were loose, some villi were broken, and the structure was destroyed. In the MP pretreatment group, the villi recovered, intestinal damage improved, and villi length was recovered from Cy‐induced oxidative stress injury. After Cy modeling, the activities of SOD, CAT, and GSH‐Px all decreased significantly, while the level of MDA increased obviously, suggesting that mice exposed to Cy suffered severe oxidative stress. Compared with the model group, preadministration of MP greatly increased SOD, CAT, and GSH‐Px activities in mice colon tissues, and reduced the MDA levels, indicating that intake of MP in advance can improve intestinal antioxidant enzymes activities, reduce the level of intestinal lipid peroxidation, and have preventive and protective effects on Cy‐induced intestinal oxidative damage. The mechanism may be through regulating the mRNA expression of antioxidant genes to improve the activity of antioxidant enzymes. Similar results have been reported in previous studies. Xue et al. found that polysaccharides from Hemp seed could significantly increase the activities of SOD, CAT, and GSH‐Px in mice serum and ileum, and reduce MDA content (Xue et al., 2020). Therefore, this study proved that MP can protect mice from Cy‐induced oxidative damage. To investigate whether MP exerts its antioxidant activity through SOD and GSH‐Px, the mRNA expression levels of antioxidant enzymes in mice colon tissues were measured. According to the results, MP upregulated the mRNA levels for SOD and GSH‐Px by a significant amount in colon tissues, these results were consistent with those in serum, suggesting that MP regulates antioxidant enzyme activities by increasing the mRNA expression levels of antioxidant enzymes in the intestinal tract, thus alleviating intestinal injury caused by oxidative stress.

**FIGURE 8:**
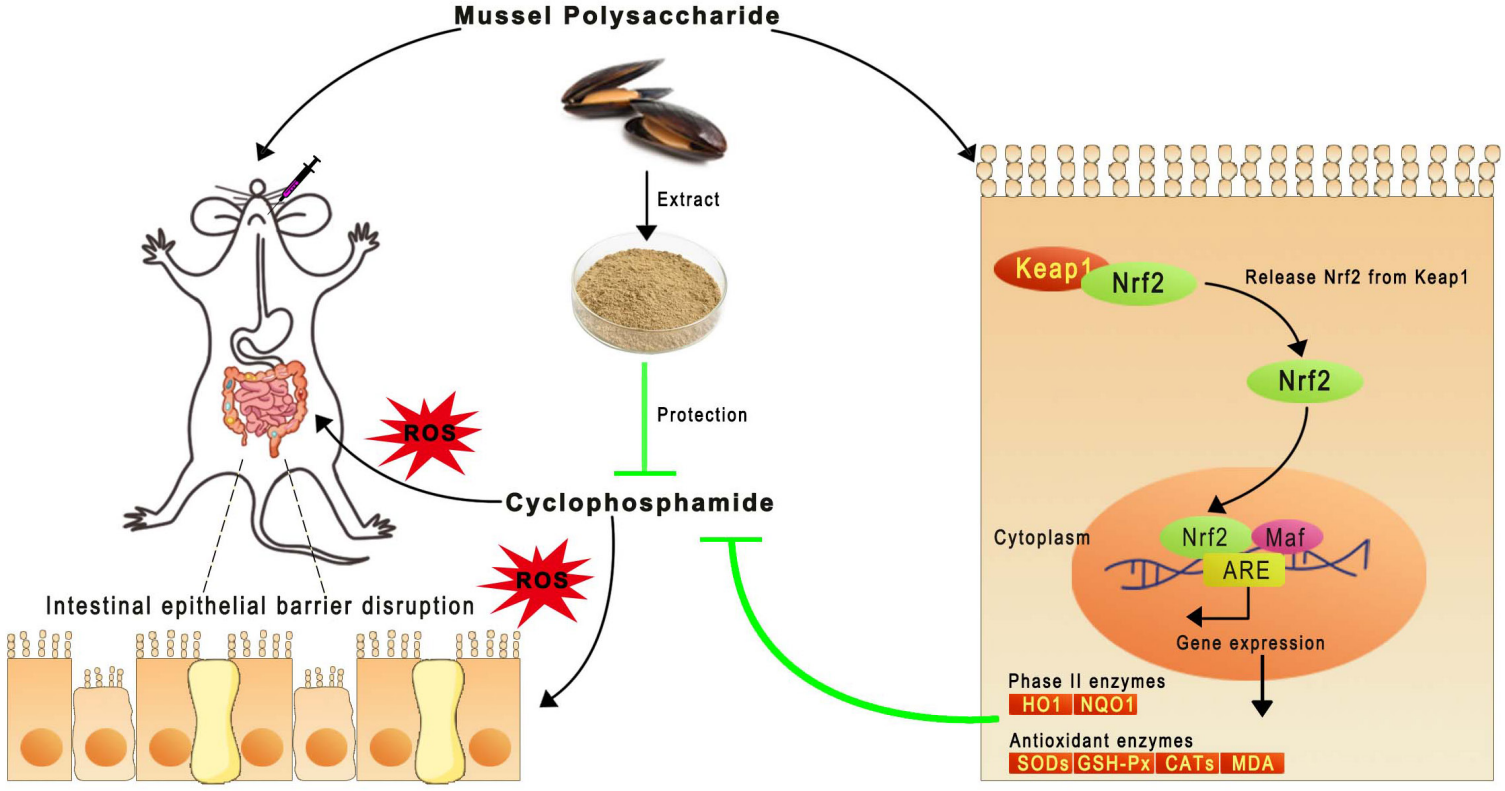
Schematic overview of mussel polysaccharides protects the intestinal of mice against cyclophosphamide‐induced oxidative stress injury via Nrf2‐Keap1 signaling pathway.

As a transcription factor, Nrf2 regulates the secretion of antioxidant enzymes in the body, thereby restoring oxidative stress damage (Chaiprasongsuk et al., 2019; Yao et al., 2019). Under basal nonstressed conditions, Keap1 binds Nrf2, generating a complex that involves Keap1‐associated ubiquitin ligases (Cul3), forming Keap1‐Cul3‐Nrf2 complex, which ubiquitinate and degrade Nrf2. In contrast, a constant accumulation of ROS under oxidative stress forces Nrf2 migrate to the nucleus and bind with antioxidant reaction elements (ARE), as a result, antioxidant enzymes and phase II detoxification enzymes are activated (Senthilkumar et al., 2006). HO‐1 and NQO1 are important downstream target genes of Nrf2‐Keap1 signaling pathway, which play key roles in oxidative stress‐mediated injury response (Yao et al., 2014). As reported previously and based on our data, MP is capable of enhancing several molecules involved in oxidative stress, suggesting that Nrf2 might be involved in this process. Our present qRT‐PCR results showed that compared with the Cy model group, MP‐treated mice showed significant increases in HO‐1 and NQO1 gene expression in Nrf2 signaling pathways in the colon tissues. Previous research also reported that *Dendrobium officinale* polysaccharide can regulate NQO‐1 and HO‐1 expressions by activating the Nrf2 core transcription factor in Cy‐injured mice (Zhao et al., 2019). Accordingly, MP may exert its antioxidant effect through the activation of Nrf2 signaling. Consequently, western blot detection of Nrf2 and Keap1 proteins was conducted. The results showed that the expression of Keap1 protein was significantly reduced and Nrf2 protein was significantly elevated in the colon tissues by MP in a dose‐dependent manner compared with the Cy model group. Moreover, Li et al. found that polysaccharides from *Ostrea rivularis* can reduce reproductive oxidative stress damage by upregulating NQO1, SOD, and HO‐1 protein expression in mice's testis via activating Keap1‐Nrf2 pathway (Li et al., 2018). These results further confirmed that MP can effectively alleviate oxidative stress injury by activating the Nrf2‐Keap1 antioxidant signaling pathway, and this mechanism is schematically represented in Figure 8.

## 5 CONCLUSION

The present study showed that SOD, CAT, and GSH‐Px levels in serum and colon tissues were significantly improved with MP, while MDA levels were significantly reduced. Further, MP pretreatment greatly enhanced SOD, GSH‐Px, Nrf2, HO‐1, and NQO1 mRNA expression in colon tissues, increased Nrf2 protein expression, and decreased Keap1 protein expression. Taken together, the above findings indicated that MP could restore and relieve the intestinal oxidative stress induced by Cy in mice, this is likely to be due to the regulation of antioxidative enzyme secretion and activation of the Nrf2‐Keap1 pathway that leads to antioxidant effects. Based on these findings, we concluded that MP has a great therapeutic potential in the treatment of several diseases related to intestinal oxidative damage.

## AUTHOR CONTRIBUTIONS

**Zhen‐Lei Zhao:** Conceptualization (equal); funding acquisition (equal); methodology (equal); writing – original draft (equal). **Xiao‐Gang Xu:** Conceptualization (equal); visualization (equal); writing – original draft (equal). **Yun‐Chuang Chang:** Investigation (equal); software (lead); writing – review and editing (equal). **Yi‐Peng Xu:** Methodology (equal); visualization (equal). **Xu‐Qiang Zhou:** Investigation (equal); writing – review and editing (equal). **Hui‐Li Su:** Formal analysis (lead); funding acquisition (equal); writing – review and editing (equal). **Xiao‐Hua Cui:** Funding acquisition (equal); resources (lead). **Xiao‐Qing Wan:** Supervision (equal). **Gen‐Xiang Mao:** Project administration (lead); supervision (equal).

## FUNDING INFORMATION

This research was funded by the project of the Research Center of Prevention and Treatment of Senescence Syndrome, School of Medicine Zhejiang University (grant number: 2022060002), Traditional Chinese Medicine Administration of Zhejiang Province (grant number: 2020ZQ002 and 2022ZQ003), National Key Research and Development Program of China (grant number: 2022YFC3501802).

## CONFLICT OF INTEREST STATEMENT

The authors declare that the research was conducted in the absence of any commercial or financial relationships that could be construed as a potential conflict of interest.

## Data Availability

The original contributions presented in this study are included in the article, further inquiries can be directed to the corresponding authors.
